# Overcoming negative predictions of microRNA expressions to gemcitabine response with FOLFIRINOX in advanced pancreatic cancer patients

**DOI:** 10.2144/fsoa-2020-0128

**Published:** 2020-11-30

**Authors:** Konstantin Schlick, Florian Hohla, Frank Hamacher, Hubert Hackl, Clemens Hufnagl, Steiner Markus, Teresa Magnes, Simon Peter Gampenrieder, Thomas Melchardt, Stefan Stättner, Cornelia Hauser-Kronberger, Richard Greil, Gabriel Rinnerthaler

**Affiliations:** 1 IIIrd Medical Department with Hematology & Medical Oncology, Hemostaseology, Rheumatology & Infectious Diseases, Oncologic Center, Paracelsus Medical University Salzburg, Müllner Hauptstrasse 48, Salzburg 5020, Austria; 2 Salzburg Cancer Research Institute with Laboratory of Immunological & Molecular Cancer Research & Center for Clinical Cancer & Immunology Trials, Salzburg, Austria; 3 Cancer Cluster Salzburg, Salzburg, Austria; 4 Division of Bioinformatics, Biocenter, Medical University of Innsbruck, Innsbruck, Austria; 5 Department of Surgery, Salzkammergutklinikum, Standort Vöcklabruck, Oberösterreich, Austria; 6 Department of Surgery, Paracelsus Medical University Salzburg, Salzburg, Austria; 7 Department of Pathology, Paracelsus Medical University Salzburg, Salzburg, Austria

**Keywords:** FOLFIRINOX, gemcitabine, miRNA, pancreatic cancer

## Abstract

FOLFIRINOX is superior to gemcitabine in patients with pancreatic cancer, but this regimen is associated with toxicity and biomarkers for response are warranted. MicroRNAs can mediate drug resistance and could provide predictive information. Altered expressions of several microRNAs including miR-21-5p, miR-10b-5p and miR-34a-5p have been previously linked to a worse response to gemcitabine. We investigated the influence of expression levels in tumor tissue of those three microRNAs on outcome to FOLFIRINOX. Twenty-nine patients with sufficient formalin-fixed paraffin-embedded tumor tissue were identified. There was no significant association between high and low expression groups for these three microRNA. We conclude that polychemotherapy combination can overcome intrinsic negative prognostic factors.

## Lay abstract

Treatment outcome with the polychemotherapy FOLFIRINOX is superior to gemcitabine in patients with pancreatic cancer, but FOLFIRINOX can cause severe toxicities. Therefore, we investigated biomarkers that may guide treatment decision. Altered expression of certain microRNAs, small noncoding RNAs, are associated with a worse response to gemcitabine. We found that the expression of the microRNAs miR-21-5p, miR-10b-5p and miR-34a-5p have no preditive value in patients treated with FOLFIRINOX. We therefore, conclude that polychemotherapy combination can overcome intrinsic negative prognostic factors.

Pancreatic cancer (PC) is the third leading cause of cancer-related death in men and women in the USA and is anticipated to take the second place in 2020 ranking second after lung cancer [1]. In 2019, 45,750 PC deaths and 56,770 new cases were estimated in the USA alone; however, by 2030, 63,000 expected cancer death from PC are predicted, making it the number one cause of cancer-related death in the next 10 years [2]. It is estimated that, by 2040, the total number of cases in the EU will increase by more than 30% [3].

PC remains one of the most devastating malignant diseases and outcome is dismal with a 5-year survival rate of only 5% [4]. A curative treatment approach with a radical surgical resection can only be offered to less than 20% of cases as most patients are either in an advanced stage of disease or present with incurable metastatic disease.

FOLFIRINOX (5-fluorouracil/leucovorin, irinotecan and oxaliplatin) is a highly active chemotherapy regimen in the adjuvant setting [5] and for the first-line treatment of patients with unresectable advanced or metastatic pancreatic cancer (APC) [6]. In a pivotal adjuvant trial, a statistically significant overall survival (OS) benefit was demonstrated for modified-FOLFIRINOX compared with gemcitabine in PC patients with an increase of 19.4 months (54.4 vs 35.0 months, stratified hazard ratio [HR] for death 0.64; 95% CI: 0.48–0.86; p = 0.003) [5]. Additionally, gemcitabine plus capecitabine led to a significant increase of OS from 25.5 to 28.0 months (HR: 0.82; 95% CI: 0.68–0.98; p = 0.032) compared with gemcitabine mono treatment in the adjuvant setting [7].

In patients with APC, FOLFIRINOX showed a significantly longer OS of 11.1 versus 6.8 months (HR: 0.57; p < 0.001) and a significant longer progression-free survival (PFS) of 6.4 versus 3.3 months (HR: 0.47; p < 0.001) compared with the former treatment standard gemcitabine in a Phase III trial including 342 patients [6]. However, this advantage in survival comes at the cost of toxicity with a significant increase of grade 3 or 4 adverse events. A dual chemotherapy combination of gemcitabine plus nab-paclitaxel was also more effective compared with gemcitabine alone as first-line treatment in APC with a significant increase of OS from 6.7 to 8.5 months (HR: 0.72; p < 0.001) and PFS from 3.7 to 5.5 (HR: 0.69; p < 0.001), respectively [7]. Based on the results of those two trials, the European Society of Medical Oncology and the American Society of Clinical Oncology recommend FOLFIRINOX or a gemcitabine plus nab-paclitaxel combination as first-line treatment in fit patients with APC [8,9].

MicroRNAs are currently under investigation in various cancer entities for their diagnostic, prognostic and predictive role as biomarkers [10]. MicroRNAs are small, approximately 22 nucleotides long noncoding single-stranded RNAs, regulating gene expression at a post-transcriptional level. The human genome may encode more than 1000 microRNAs and approximately 60% of human genes are regulated by microRNAs. Besides other functions, they are known to be involved in tumor evolution [11] including regulation of angiogenesis [12] and development of treatment resistance [13]. However, no existing microRNA panel is currently endorsed for clinical use in therapy response. Altered microRNA expression profiles were described in different malignancies [14]. Generally, microRNAs act as tumor suppressors, by negatively regulating oncogenes, genes that promote cell proliferation, as well as genes that inhibit cell division [15,16]. Therefore, dysregulation can lead to carcinogenesis with loss of normal function resulting in altered expression of, for example, tumor suppressor genes. Based on these findings, it is not surprising that microRNAs can be used for tumor diagnosis and can provide both prognostic and predictive information in different cancer entities.

MicroRNA can be analyzed from several body fluids like blood, but also from tissue specimen. Although microRNA analysis from blood samples has the advantage of being noninvasive and samples are easily accessible with a minimum risk for the patient, the measured microRNAs are not cancer and disease specific [17–19]. In contrast, microRNA analyses from tumor tissue specimen allow better conclusions to be drawn about tumor-associated expressions. Additionally, archived tumor tissue is commonly available compared with archived blood samples, which are rare. Therefore, unplanned retrospective analysis is more feasible from tumor tissue.

In PC, a distinct microRNA expression profile compared with benign lesions has been observed [20–24]. Using microRNA expression signatures consisting of 20 to 32 microRNAs, a clear discrimination between healthy, inflamed and cancerous pancreatic tissue can be made [20,22,24]. These findings are of clinical relevance, because histological clarification of pancreatic lesions is often challenging, particularly in tissues obtained by endo-sonography-assisted fine-needle biopsies. Furthermore, plasma levels of circulating miR-221 are significantly higher in samples from patients with PC compared with healthy controls and the expression level is correlated with prognosis [25].

Beyond the diagnostic value of microRNA expression profiles, several microRNAs like miR-155, miR-21, let7, miR200c, miR34a have shown to be prognostic and predictive [20,26–35].

The predictive value of microRNAs for therapy response under gemcitabine in PC patients was shown *in vitro* and *in vivo*. An altered expression of miR-21 (overexpression), miR-10b and miR-34a (downregulation) has been previously associated with worse survival under gemcitabine chemotherapy [29–31,36,37]. The aim of this study was to investigate the influence of those three microRNAs on the treatment outcome of FOLFIRINOX in patients with APC and whether FOLFIRINOX can overcome these negative predictors to gemcitabine treatment.

## Materials & methods

### Study population

In this single center study at the IIIrd Medical Department of the Paracelsus Medical University Salzburg, consecutive patients with advanced PC eligible for treatment with FOLFIRINOX between 2010 and 2012 were retrospectively analyzed. All patients treated with at least one dose of FOLFIRINOX as first-line treatment, an age over 18 years and sufficient available tumor tissue for microRNA analysis were eligible for our analyses.

Dose modifications were made at the discretion of the treating physician. As per clinical standard, performance status and comorbidities where considered for primary dose modifications. Furthermore, treatment-associated toxicities resulted in secondary dose modifications as decided by the investigator. As per our institutional standard, treatment was continued until disease progression or documentation of unacceptable toxicities. We retrospectively evaluated patient characteristics, Eastern Cooperative Oncology Group (ECOG) performance score [38], date of diagnosis, start of FOLFIRINOX treatment, dose modifications during treatment, toxicity, response rates, PFS and OS based on review of patient’s medical records and radiology reports. Tumor response was defined by using CT scans classified into partial response, stable disease or progressive disease according to WHO criteria taking tumor marker response into account [39].

### Tissue samples

Formalin-fixed paraffin-embedded (FFPE) tissue samples from primary tumors, or if available, from metastatic sites, were selected by an experienced pathologist (C Hauser-Kronberger). All tissue samples were collected prior to the start of first-line chemotherapy for metastatic disease. Three to five 10-μm paraffin-sections were cut from each block without micro- or macro-dissection and placed in sterile Eppendorf tubes.

### RNA purification & microRNA expression analysis

Total RNA was purified from FFPE-Tissue using the *mir*Vana™ microRNA Isolation Kit from Ambion^®^. The concentration and purity of total RNA was determined by measuring 1.5 μl from RNA isolation with NanoDrop (ThermoFisher Scientific, MA, USA) and 1 μg was reversely transcribed to cDNA using the TaqMan^®^ Reverse Transcriptase Kit and specific reverse PCR primers (*TaqMan MicroRNA Assay*) according to the manufacturer’s instructions. (For microRNA target sequence see Supplementary Table 2) MicroRNA expression was quantified using microRNA-specific forward primers (*TaqMan MicroRNA Assay*) for hsa-miR-21-5p, hsa-miR-10b-5p, hsa-miR-34a-5p and RNU6b in combination with the *TaqMan Universal PCR Master Mix* by quantitative real-time PCR.

### Statistical analysis

PFS was defined as the time from start of FOLFIRINOX treatment until date of confirmed progression or death from any cause. OS was as defined from start of FOLFIRINOX treatment until death from any cause. Data analysis for this retrospective study was descriptive in nature and presented in medians and ranges (95% CI). PFS and OS estimates were obtained using the Kaplan–Meier (KM) method. The median follow-up duration was measured by reverse KM estimator. Comparing survival curves by the use of Cox regression univariate analyses including HR with 95% CI. The HR describes the relative risk of the occurrence of an end point of interest based on comparison of event rates between patient groups. The HR is equivalent to the odds that an individual in the group with the higher hazard reaches the end point first. The association of microRNA expression, dichotomized based on median expression, with PFS or OS was analyzed for each microRNA using a log rank test.

Comparisons of microRNA expressions between adjuvant gemcitabine treatment groups were performed using the Wilcoxon rank sum exact test.

All statistical analyses were performed using the statistical software environment R (packages survival). A p-value of <0.05 was considered to be statistically significant with regard to median OS with 95% CI.

### Ethics

This analysis was approved by the Ethics Committee of the federal state of Salzburg, Austria (IRB number: 415-EP/73/236-2013). Due to the retrospective character, the pseudonymized analysis and the death of all patients at the time point of data analysis, the committee waived the requirement for informed consent.

## Results

**Figure 1.**
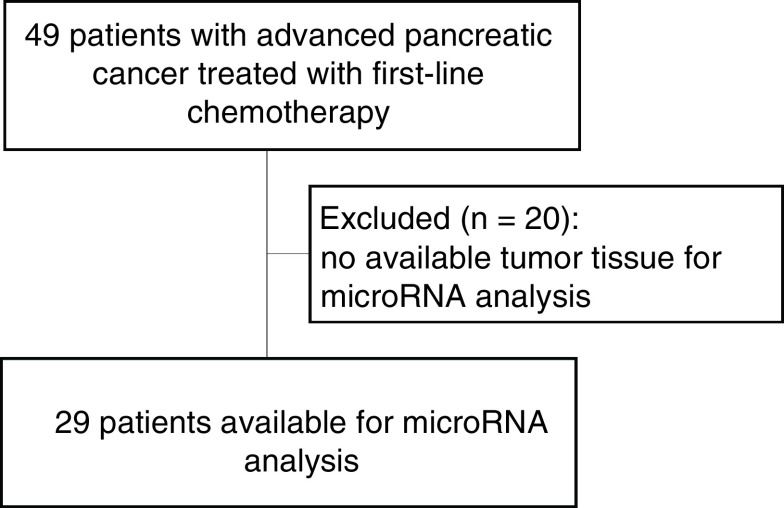
Consort diagram.

Forty-nine patients with APC treated between 2010 and 2012 at the IIIrd Medical Department of the Paracelsus Medical University Salzburg with first-line FOLFIRINOX were identified. In twenty-nine patients (59% of the study cohort), sufficient FFPE tumor tissue was available for our analyses (Figure 1). Median age at diagnosis was 63 years (45–75 years), 14 (48%) were male and the majority of our female and male study cohort had an ECOG performance status of 0 or 1 as expected for a selection for polychemotherapy. Patient’s baseline characteristics are outlined in Table 1. Forty-two samples derived from primary tumor and 16 (28%) from metastasis (3 lymph node, 3 liver, 2 lung, 1 pleural, 2 soft tissue, 3 skin, 1 ovary, 1 bone marrow). Twenty-two (38%) specimens were achieved by core biopsy and 36 (62%) by surgery.

**Table 1.** Patient characteristics with available tumor tissue.

Characteristic	n = 29
Age	
– Median	63
– Range	45–75
Gender	
– Female	15 (52%)
– Male	14 (48%)
Grading	
– G2	20 (69%)
– G3	8 (28%)
– Unknown	1 (3%)
Histology	
– Adenocarcinoma	28 (97%)
– Adenosquamous carcinoma	1 (3%)
Gemcitabine treatment	
– Adjuvant	8 (28%)
– After progression on FOLFIRINOX	11 (38%)
Relapse or primary advanced	
– Primary locally advanced non-metastatic	3 (10%)
– Primary metastatic	15 (52%)
– Unresectable relapse after initial resection	11 (38%)

Overall median PFS (95% CI) and median OS (95% CI) for the whole study cohort was 4.67 (3.48–7.73) and 10.52 (9.66–13.12) months, respectively.

Upon purification of RNA, median RNA concentration was 0.09 μg/μl (0.02–0.83) with median sample purity of 1.65 (1.10–2.87; 260/280 nm ratio) and 1.17 (0.07–6.34; 250/230 nm ratio).

**Figure 2.**
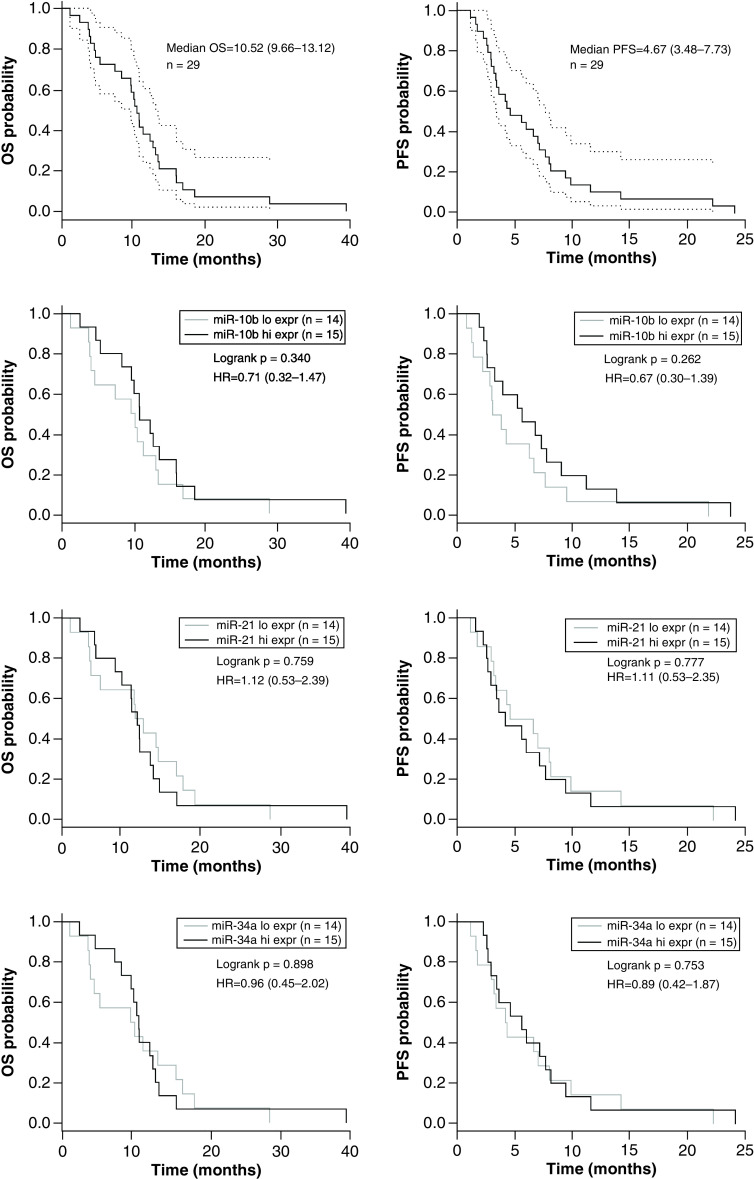
Overall and progression-free survival Kaplan–Meier plots for the overall population and for the microRNA expression groups (high vs low) of miR-21-5p, miR-10b-5p and miR-34a-5p. Median OS and median PFS are shown for overall study population including CIs in the first row. In the following blots median OS and median PFS based on the median dichotomized microRNA expression groups (high vs low) are illustrated for miR-21-5p, miR-10b-5p and miR-34a-5p. Hi expr: High expression; HR: Hazard ratio; Low expr: Low expression; OS: Overall survival; PFS: Progression-free survival.

Patients were divided into a low (n = 14) and high (n = 15) expression group, based on the median expression level of the microRNA in the tumor. MicroRNA expression levels are listed in Supplementary Table 1. There was no significant association between high and low expression groups with miR-21-5p (HR = 1.1; p = 0.78; HR = 1.12; p = 0.76), miR-10b-5p (HR = 0.67; p = 0.26; HR = 0.71; p = 0.34) and miR-34a-5p (HR = 0.89; p = 0.75; HR = 0.96; p = 0.90), with regard to PFS and OS, respectively (see Figure 2 for KM plots & Supplementary Figure 1 for correlation analyses). No statistically significant differences of the expressions of miR-21 (p = 0.55), miR-10p (p = 0.37) and miR-34a (p = 0.79), were seen between patients treated with adjuvant gemcitabine compared with patients with no adjuvant gemcitabine therapy.

## Discussion

To our knowledge, this is the first study to demonstrate that FOLFIRINOX polychemotherapy can overcome the poor predictive value of certain microRNAs shown in APC patients treated with gemcitabine. With higher response rates and lower HRs for recurrence and death, FOLFIRINOX seems to be more effective but also more toxic than the gemcitabine combination. Grade 3–4 side effects occur quite frequently, which raises questions of patient selection and leads to dose-reduction and dose-modified schedules. Proper patient selection is crucial to identify those that are most likely to benefit from aggressive chemotherapy approaches and separate those, who will likely have only little benefit due to increased rates of severe side effects. However, no prospectively validated models are available to guide decision making for an upfront patient selection.

MiR-21 is commonly considered an oncogene and exhibits anti-apoptotic activity. It enhances cellular proliferation, migration and invasion, leading to aggressive tumor progression. *In vitro* studies suggested that miR-21 conferred chemoresistance via modulation of apoptosis, Akt phosphorylation and expression of genes involved in cellular invasiveness [17]. Further miR-21 confers chemoresistance by targeting FAs-ligand and inhibiting gemcitabine-induced apoptosis. Furthermore miR-21 induces 5-fluorouacil resistance in human pancreatic cells by regulating PTEN and PDCD4 published by Wei *et al.* [40]. Hwang *et al.* showed a negative impact of miR-21 in PC patients treated with Gem or 5-FU containing adjuvant regimen [30]. Therefore, an altered expression of miR-21 (overexpression), miR-10b and miR-34a (downregulation) has been previously associated with worse survival under gemcitabine chemotherapy [29–31,36,37].

In our analyses, no influence by the expression levels of miR-21-5p-, miR-10b-5p and miR-34a-5p on PFS and OS could be detected. This suggests that these mRNAs, in contrast to gemcitabine, do not play a role for FOLFIRINOX efficacy. With polychemotherapy, one might overcome negative prediction of certain mRNA to single agent therapy.

Despite the investigation of miRNA in tumor tissue, expression profiles of circulation miRNAs have also been investigated as potential biomarkers for PC. Meijer *et al.* demonstrated that a decline in plasma miR-181a-5p levels after 5–6 cycles of FOLFIRINOX was associated with better prognosis and may be useful for guiding therapeutic choices and surgical exploration [41]. This association was not observed in a second cohort of patients treated with gemcitabine plus nab-paclitaxel. *In vitro* analyses detected an increased sensitivity of PC cells lines to oxaliplatin when miR-181a-5p was inhibited.

Several limitations should be considered in the present study. First, the rather small sample size precludes a definitive conclusion about the predictive and prognostic value of the investigated miRNAs. Furthermore, the lack of a control group not treated with FOLFIRINOX is a further limitation. However, our study does not provide any evidence that the three investigated micro-RNAs do have any influence on the efficacy of FOLFIRINOX as first-line therapy of APC.

## Future perspective

Expression levels of miR-21-5p, miR-10b-5p and miR-34a-5p in pancreatic tumor tissue do not predict treatment outcome to FOLFIRINOX. As altered expressions of these microRNAs were shown to be associated with a worse response to gemcitabine, these markers may help to select patients for a treatment with FOLFIRINOX or a gemcitabine-based regimen, respectively. Hopefully in the near future more biomarkers will arise and be validated for novel therapeutic agents like nab-Paclitaxel or nano-liposomal-irinotecan in order to properly select patients to certain personalized chemotherapeutic regiment.

**Summary points**

- FOLFIRINOX is superior to gemcitabine in patients with pancreatic cancer (PC), but this polychemotherapy regimen is associated with more toxicities.
- Biomarkers guiding treatment decision for PC patients are lacking.
- Tissue expression of several microRNAs like miR-21-5p, miR-10b-5p and miR-34a-5p have been previously linked to worse outcome in gemcitabine treatment patients with PC.
- In this retrospective analysis of patients treated with FOLFIRINOX, miR-21-5p, miR-10b-5p and miR-34a-5p tissue expressions levels were not correlated with outcome.
- We conclude that polychemotherapy combination can overcome intrinsic negative prognostic factors.
