# Supplementary material for: Overcoming negative predictions of microRNA expressions to gemcitabine response with FOLFIRINOX in advanced pancreatic cancer patients
Source: Future Sci OA. 2020 Nov 30;7(2):FSO644. doi: 10.2144/fsoa-2020-0128 (PMC7787156; doi:10.2144/fsoa-2020-0128)
Supplement: Supplementary file 1 [file fsoa-07-644-s1.docx]

**Supplemental material**

Fig S1: Correlation analyses for miR-21-5p; miR-10b-5p; miR-34a-5p in regard to overall survival and progression free survival


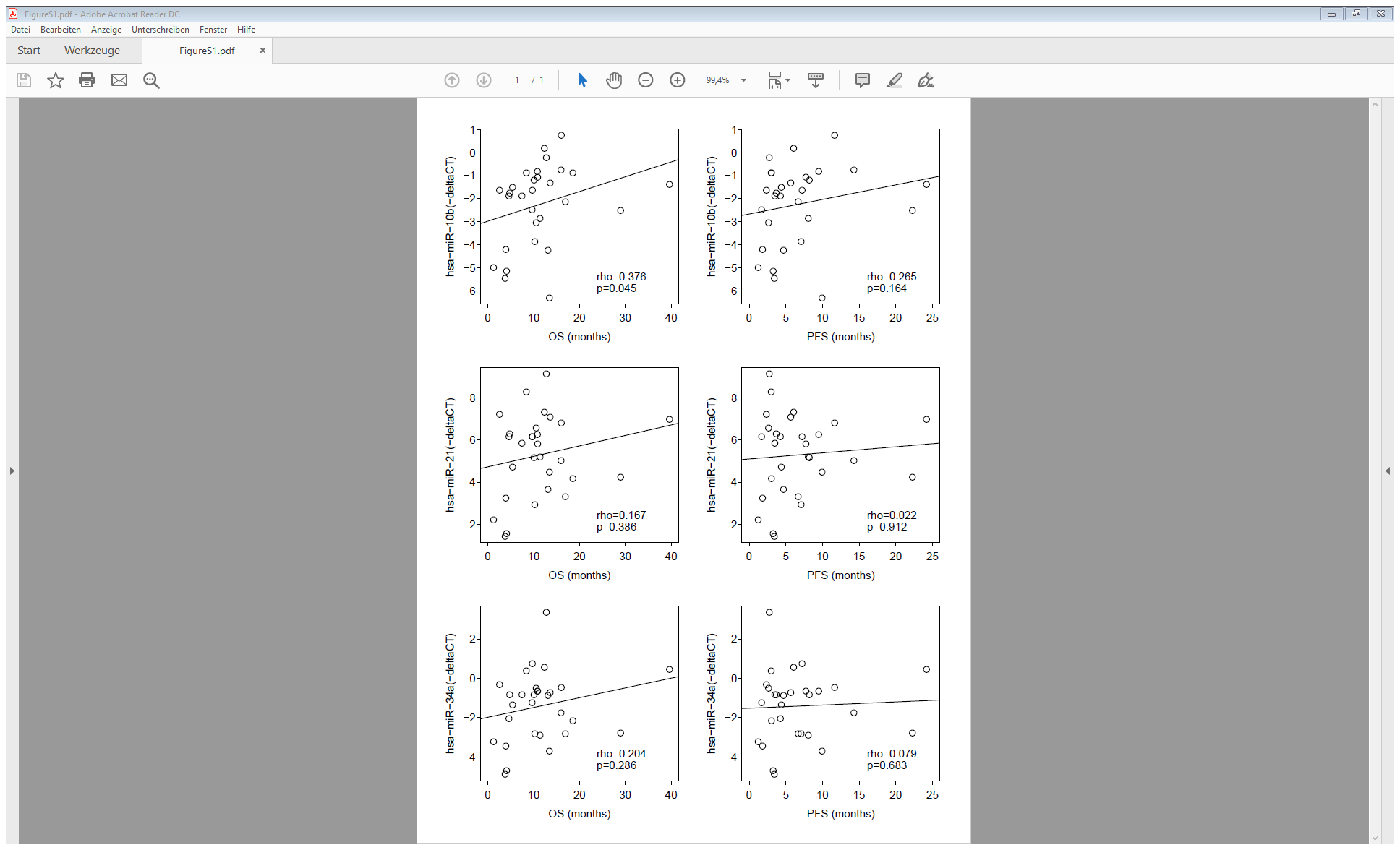


Tab S1: median expression levels for miR-21-5p; miR-10b-5p; miR-34a-5p in 29 patients

in regard to overall survival and progression free survival

| **LAB_ID** | **OS** | **PFS** | **miR-10b** | **miR-21** | **miR-34a** |
| --- | --- | --- | --- | --- | --- |
| 1 | 13,445 | 9,928 | -6,299 | 4,490 | -3,701 |
| 2 | 11,374 | 8,054 | -2,866 | 5,195 | -2,886 |
| 5 | 16,897 | 6,673 | -2,142 | 3,319 | -2,821 |
| 6 | 12,755 | 2,728 | -0,217 | 9,150 | 3,359 |
| 7 | 7,462 | 3,485 | -1,892 | 5,858 | -0,824 |
| 8 | 13,116 | 4,668 | -4,239 | 3,667 | -0,883 |
| 9 | 9,730 | 7,199 | -1,636 | 6,160 | 0,739 |
| 10 | 10,092 | 8,185 | -1,188 | 5,151 | -0,836 |
| 12 | 10,848 | 7,725 | -1,063 | 5,822 | -0,631 |
| 13 | 5,391 | 4,372 | -1,505 | 4,712 | -1,363 |
| 14 | 1,216 | 1,216 | -5,003 | 2,214 | -3,223 |
| 15 | 4,602 | 4,241 | -1,895 | 6,176 | -2,030 |
| 16 | 9,665 | 1,677 | -2,490 | 6,173 | -1,247 |
| 17 | 10,815 | 9,467 | -0,806 | 6,253 | -0,647 |
| 18 | 39,612 | 24,162 | -1,362 | 6,999 | 0,464 |
| 19 | 28,961 | 22,255 | -2,522 | 4,239 | -2,778 |
| 20 | 4,767 | 3,682 | -1,767 | 6,284 | -0,828 |
| 21 | 2,564 | 2,334 | -1,615 | 7,213 | -0,332 |
| 22 | 8,383 | 2,991 | -0,869 | 8,302 | 0,385 |
| 23 | 13,609 | 5,654 | -1,323 | 7,101 | -0,736 |
| 24 | 10,519 | 2,630 | -3,055 | 6,563 | -0,498 |
| 25 | 4,076 | 3,254 | -5,133 | 1,566 | -4,703 |
| 26 | 3,912 | 1,808 | -4,192 | 3,250 | -3,459 |
| 27 | 10,224 | 7,068 | -3,845 | 2,925 | -2,814 |
| 28 | 16,009 | 11,637 | 0,753 | 6,800 | -0,474 |
| 29 | 12,327 | 6,049 | 0,186 | 7,321 | 0,555 |
| 30 | 3,748 | 3,419 | -5,464 | 1,437 | -4,892 |
| 31 | 18,540 | 3,024 | -0,887 | 4,175 | -2,173 |
| 32 | 15,976 | 14,267 | -0,735 | 5,030 | -1,748 |

Tab S2: Target sequence of investigated microRNAs

| **microRNA name** | **miRBase ID (v21)** | **Assay Target Sequence** |
| --- | --- | --- |
| miR-21-5p | hsa-miR-21-5p, | UAGCUUAUCAGACUGAUGUUGA |
| miR-10b-5p | hsa-miR-10b-5p | UACCCUGUAGAACCGAAUUUGUG |
| miR-34a-5p | hsa-miR-34a-5p | UGGCAGUGUCUUAGCUGGUUGU |
